# Self- and informant-reported cognitive concerns associated with primary brain tumour: systematic review

**DOI:** 10.1007/s00520-025-09345-5

**Published:** 2025-03-21

**Authors:** Shelley Mekler, Sian Virtue-Griffiths, Kerryn Pike

**Affiliations:** 1 School of Applied Psychology, Griffith Health, Griffith University, 1 Parklands Drive, Gold Coast, QLD Australia; 2 School of Psychology & Public Health, La Trobe University, Melbourne, VIC Australia; 3 Griffith Centre for Mental Health, Griffith University, Gold Coast, Australia

**Keywords:** Primary brain tumour, Subjective cognitive concern, Informant, Cognition, Neuropsychological assessment

## Abstract

**Purpose:**

People with primary brain tumour (PBT) experience objective cognitive impairment, but subjective cognitive concerns have received less attention. This review sought to determine the type of self- and informant-reported cognitive concerns following PBT and to ascertain if they vary according to patient, tumour and/or treatment characteristics. Further objectives were to determine whether subjective reports aligned with objective findings or informant reports.

**Methods:**

Literature searches were conducted using PsycINFO and Medline, limited to English-language and full-text format. Studies focusing on non-PBTs, objective cognition without subjective reports, or paediatric PBT were excluded.

**Results:**

Eleven studies were included, representing the cognitive concerns of 957 PBT participants, with varying tumour types/treatment, and ranging from pre-surgery to an average of 5 years post-diagnosis. Subjective concerns regarding global perceived cognitive impairment, language, memory, executive function, and attention were common, but change in processing speed, visual function, and reading/spelling were also reported. Few studies investigated factors impacting subjective cognition, but there was some suggestion that left-lateralised and larger tumours resulted in more subjective concerns. The alignment between objective and subjective cognition varied, ranging from strong to weak, whereas the overlap between patient and informant reports was robust.

**Conclusions:**

Identifying the alignment between patient and informant reports is of significant benefit when considering treatment interventions and outcomes for people with PBT, particularly in instances where they may not be able to report their cognitive concerns. Overall, the importance of the patient perspective was highlighted, which can often be replaced by objective measures in clinical research.

## Background

Cognitive impairment is a significant challenge for people with primary brain tumour (PBT), and often impacts daily functioning [1]. Up to 49% of people with PBT exhibit objective cognitive deficits [2], which frequently relate to memory, verbal function, attention, executive function (EF), or global cognitive decline [3]. The extent and nature of objective cognitive impairment varies depending on tumour characteristics, disease prognosis, and treatment modalities [4–7]. These impairments are associated with reductions in quality of life, hindering individual, family, and social functioning [8].

Comprehensive neuropsychological test batteries are commonly used to conduct an examination of cognition [9]. These examinations reveal the prevalence of cognitive impairment for people with PBT, facilitate early detection of cognitive impairment, and aid in the formulation of effective interventions [7–10]. These specialised assessments are not always available in clinical practice [11], however, and subjective report would provide some indication of the impact of cognitive difficulties, which can be underestimated or overlooked by clinicians [12]. Moreover, the importance of ascertaining subjective cognition for cognitive rehabilitation has been highlighted by multiple researchers [13–15], despite acknowledgement that there exists a lack of treatments considering subjective cognitive concerns [15–17]. Although limited research has investigated the self-reported cognitive concerns of people with PBT, there is some indication that the association between objective and subjective measures of cognitive difficulties is only weak to moderate [18, 19]. This raises the possibility that the difference between objective assessment and subjective reports is due to a lack of patient insight and may warrant the evaluation of informant reports. Alternatively, some people with PBT may notice changes in their cognition that are too subtle to be detected by objective tasks, yet still cause challenges in their daily lives.

This review sought to identify the type and prevalence of subjective cognitive issues for people with PBT and determine which domains are most frequently regarded as impaired. A secondary objective was to ascertain whether, and to what degree, subjective cognitive concerns differ according to patient characteristics (age, gender, level of education), brain tumour characteristics (histology, grade, location, time since diagnosis), and treatment characteristics (treatment modality, time since treatment). Additionally, the alignment between subjective cognitive concerns and the findings of neuropsychological tests, as well as the congruency of self-report with informant report, were also investigated.

## Method

### Protocol registration

This study was conducted following the Preferred Reporting Items for Systematic Reviews and Meta-analyses (PRISMA) guidelines [20]. The study protocol was pre-registered on Open Science Framework (OSF) on August 27, 2022 (https://doi.org/10.17605/OSF.IO/UGS6A). No amendments were made to the registered protocol.

### Eligibility criteria

Studies were deemed eligible for inclusion if participants were people with PBT, older than 18 at the time of baseline examination, and included a subjective report addressing cognitive issues. Studies were excluded if they did not measure cognitive difficulties linked to PBT, presented only objective measurements, did not give access to the full text, did not report primary data, or were published in non-English language. Further, studies were excluded if participants were diagnosed with a paediatric PBT, due to differences between paediatric and adult PBTs regarding histology, molecular and genetic subgroups, incidence, and prognoses [21]. Finally, studies assessing social cognition only were excluded.

### Search strategy

To identify eligible articles, PsycINFO and MEDLINE were searched up to 23 August 2022. Keyword searching was used for 3 sets of terms (brain cancer, subjective, and cognitive impairment), using “mapped to subject heading” and synonyms for each key term. The following key terms were used:

1. Brain Cancer OR Malignant Brain Tumor OR Brain Tumor OR Primary Brain Tumor OR Brain Neoplasm OR Glioma
  AND
2. Self-Report OR Subjective
  AND
3. Cognitive Impairment OR Cognitive Decline OR Cognitive Dysfunction OR Mild Cognitive Impairment OR Executive Dysfunction

### Study selection

Covidence (Veritas Health Innovation) was used for study selection and data extraction. After removal of duplicates, titles and abstracts were examined by one reviewer (first author) to assess eligibility. The full texts of prospective eligible papers were subsequently evaluated independently by two authors (first author and second author). The degree of agreement was investigated, and differences were resolved through consensus. The same two authors then independently applied the JBI Prevalence Studies Critical Appraisal Tool to assess key methodological aspects and ensure the credibility and reliability of the evidence summarised in our review.

### Data extraction

One reviewer (first author) independently extracted data from all included publications using a data collection form that was previously tested on a sample of two studies selected at random. The following data were extracted:

- Which cognitive domains are reported by patients and informants as impaired?
- Which cognitive domain is reported as the most impaired?
- Potential moderators: data concerning factors which may influence the impaired cognitive domain, including sample characteristics (age, gender, education), disease characteristics (tumour histology, tumour lateralisation, time since diagnosis, treatment modality, time since treatment), and study characteristics (measure(s) used to assess subjective cognitive impairment, total number of participants, objective measures, inclusion and exclusion criteria)
- Alignment of subjective report with findings of objective neuropsychological tests
- Alignment of self-report with informant report

## Results

### Selection of studies

**Fig. 1:**
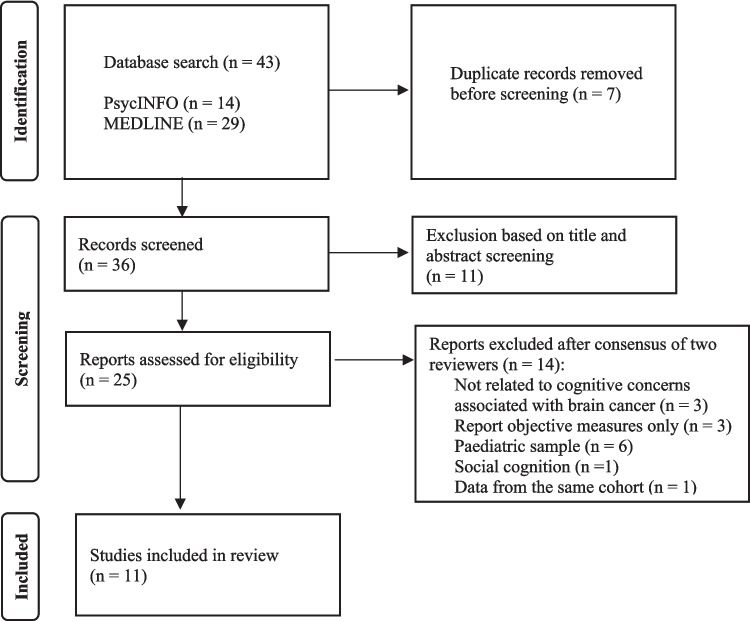
PRISMA flow chart illustrating the process of study selection

The initial literature search produced 43 articles, which were reduced to 36 following the removal of duplicate papers. A further 11 were eliminated based on their title and abstract, leaving 25 articles to be reviewed as full texts. There was 80% agreement between the two raters on the eligibility of the 25 remaining citations, with discrepancies settled through consensus. Thus, the systematic review comprised 11 studies in which data was extracted. Figure 1 illustrates the PRISMA flow chart in further detail.

### Study characteristics

The 11 included studies provided information on 957 participants’ cognitive concerns associated with PBT. The descriptive characteristics of the included studies are shown in Table 1. Four studies came from the United States of America (USA), three from the Netherlands, and one from each of Australia, Lithuania, Germany, and Norway. With sample sizes ranging from 15 to 237, participants had an average baseline age of 51 (range 18–83), with females representing 41% overall. The studies included a range of tumour types, with the most prevalent being astrocytoma, oligoastrocytoma, and glioblastoma. Surgery, chemotherapy, and radiation therapy were frequently reported as treatment modalities. Each study reported either average time since diagnosis, or time since treatment, none reported both. Only four studies reported average time since tumour diagnosis, which ranged from 20 months to over 5 years. The other studies reported time since treatment—three were conducted prior to surgery (one also evaluating approximately 7 months later), 1 during chemoradiation therapy, and three others reported average time since treatment, which ranged from 1 month to 5 years.

**Table 1:** Descriptive characteristics of included studies

First author (year)	Country	Total *N*	Age (*M)* at the time of the study	Gender (%F)	Time since diagnosis *M* (years)	Time since Tx *M* (years)	Tumour type (%)	Tumour location (%)	Treatment type (%)^a^
Armstrong (2012) [22]	USA	115	48.2	37	1.7	NR	ASTR/OLI (33.9) Other (30.3)	L (55.7) R (38.3) MID (3.5)	CT (27.8) RT (10.4)
Braun (2021) [23]	USA	102	49.0	47	2.8	NR	ASTR (23.5) GBM (44.1) Other (32.4)	L (46.1) R (40.2) BI (10.8)	CT (49) RT (49) Res (76.5) Bx (23.5)
Cubis (2019) [24]	Australia	70	51.3	60	5.1	NR	BN (45.7) LGG (18.6) HGG (35.7)	NR	RC (1.4) SC (2.9) Su (21.4) SRC (34.3) SR (17.1) SxM (22.9)
Donix (2022) [25]	Germany	15	50.0	40	NR	Currently receiving chemoradiotherapy	ASTR 26.7% OLI 6.7% GBM 66.7%	L (26.7) R (66.7) MID (6.7)	RT (100) CT (100)
Ediebah (2017) [26]	Netherlands	195	40.8	39	5.6	NR	ASTR 71% OLI 22% OA 7%	L (49.74) R (46.67) BI (3.59)	RT (53.33) Res (56.92) Bx (43.08)
Klein (2001) [2]	Netherlands	68	54.4	22	NR	0.1	AA (52.9) OLI (10.3) GBM (36.8	L (42.6) R (55.9) BI (1.5)	Bx (16) Res (84)
Pranckeviciene (2017) [18]	Lithuania	62	58.3	60	NR	Pre-surgery	GL (32.3) MEN (50.0) Other (17.7)	L (40.3) R (53.4) MID/BI (9.7)	None (pre-treatment)
Racine (2015) [27]	USA	22	39.8	59	NR	Pre-surgery; 0.59	LGG (100)	L (91) R (9)	Su (100)
Schei (2022) [28]	Norway	237	61.0	35	NR	Pre-surgery	LGG (21) HGG (79)	L (49) R (49) BI (2)	None (pre-treatment)
van der Linden (2020) [19]	Netherlands	47	51.0	55	NR	0.25	MEN (60) LGG (40)	NR	Res (100)
Wang (2022) [29]	USA	24	44.5	33	NR	5.1	DF (100)	L (50) R (50)	SRC (95.8) Su (4.16)

^a^Percentages do not always add up to 100% as in some cases treatments were overlapping. AA anaplastic astrocytoma; ASTR astrocytoma; BI bilateral; BN benign glioma; Bx biopsy; CT chemotherapy; DF diffuse glioma; GBM glioblastoma; GL glioma; HGG high-grade glioma; L left; LGG low-grade glioma; MEN meningioma; MID midline; N number of participants; NR not reported; OA oligoastrocytoma; OLI oligodendroglioma; R right; Res resection; RC radiation therapy and chemotherapy; RT radiation therapy; SC surgery and chemotherapy; SR surgery and radiation therapy; SRC surgery, radiation therapy, and chemotherapy; Su surgery; SxM monitoring/symptom management; Tx treatment; USA United States of America

### Reported subjective cognitive difficulties

To answer our first research question regarding the type of subjective cognitive concerns reported, as shown in Table 2, the included studies reported concerns in global cognitive measures, memory, comprehension, language, attention, EF, visual function, reading/spelling, and processing speed. There was a great deal of variation, however, in (a) the number of items used in each domain; (b) the assessment tools used to measure each domain; and (c) which domains were evaluated. In terms of number of items used per domain, several studies identified cognitive concerns based on only one question per cognitive domain [22, 26–28], whereas other studies used entire questionnaires [19, 23]. Regarding tools used, except for the Behaviour Rating Inventory of Executive Functioning for Adults (BRIEF-A/IR) [30] and the Functional Assessment of Cancer Therapy – Cognition (FACT-Cog) [31], which were each administered by two studies, all other assessment measures varied. In terms of domains assessed, many studies measured multiple cognitive [16, 22, 25, 27, 28], although which domains varied. Three studies focused on a general measure of cognitive impairment without specifying which domains were impaired [2, 24, 29], and another only reported results across combined domains [28]. One study looking at general cognitive impairment also included a separate measure of language [2]. Another study focused on language only [26], albeit with only one question to ascertain language ability, and two additional studies limited their investigation to EF [19, 23].

**Table 2:** Methodology of included studies

First author (year)	Subjective measure	Subjective measure design	Cognitive domains assessed	Cognitive domains reported as reduced	Informant report (Y/N)	Objective measure (Y/N)
Armstrong [22]	MDASI-BT	28-item questionnaire (4-item assess cog. function)	Memory, language, concentration, comprehension	Memory (48.7%), comprehension (33.6%), language (32.4%), attention (26.5%)	Y	Y
Braun [23]	BRIEF-A; BRIEF-IR	75-item questionnaire (A) 75-item questionnaire (IR)	Inhibitory control, planning/organisation, working memory, global executive functioning	NR	Y	Y
Cubis [24]	FACT‐Cog	37-item questionnaire (20-item PCI used)	Global PCI	PCI	N	Y
Donix [25]	FLei	30-item questionnaire (30-item assess cog. function)	Memory, attention, EF	NR	N	Y
Ediebah [26]	QLQ-BN20	20-item questionnaire (1-item assessing cog function)	Communication	NR	Y	Y
Klein [2]	MOS	6-item questionnaire (6-item assess cog. Function)	Memory, attention, EF, processing speed (to obtain a global PCI rating)	Global PCI	N	Y
Pranckeviciene [18]	BQ-AV	31-item questionnaire (31-item assess cog. function)	Memory, EF, attention, language	Memory (8–63%), EF (13–52%), attention (19–58%), language (10–58%)	N	Y
Racine [27]	NI	N/A	Memory, attention, language, processing speed, EF, reading/spelling	Memory (46–68%), language (73–77%), attention (15–36%), EF (31–32%), processing speed (8–9%), visual function (15–18%), reading/spelling (18–31%)	Y	Y
Schei [28]	EORTC QLQ-C30	50- item QOL questionnaire (2-item assess cog. function)	Memory, attention (for global measure)	49.8% report clinically important reduced cognition (memory + attention) compared to 23.4% in reference group	N	N
van der Linden [19]	BRIEF-A; BRIEF-IR	75-item questionnaire (A) 75-item questionnaire (IR)	Inhibitory control, planning/organisation, working memory, global executive functioning	Metacognition index	Y	Y
Wang [29]	FACT-Cog	37-item questionnaire (20-item PCI used)	Global PCI	PCI	N	Y

BQ-AV Background Questionnaire – Adult Version by Strauss, Sherman, and Spreen (2006); BRIEF-A Behaviour Rating Inventory of Executive Function – Adult; BRIEF-IR Behaviour Rating Inventory of Executive Function – Informant Reported; EORTC QLQ-C30 European Organisation for Research and Treatment of Cancer Core Quality of Life questionnaire; FACT‐Cog Functional Assessment of Cancer Therapy‐Cognitive Function; FLei questionnaire for complaints of cognitive disturbances; FrSBe Frontal-Systems Behaviour Scale; MDASI-BT MD Anderson Symptom Inventory-Brain Tumour; MOS Medical Outcomes Study; N no; NI neuropsychological interviews; NR not reported; PCI perceived cognitive impairment; QLQ-BN20 Quality of Life Questionnaire BN20; Y yes

This variability in which domains were assessed made it difficult to answer our research questions regarding the prevalence and frequency of subjective concerns in different cognitive domains. Furthermore, some of the studies did not report whether people with PBT had greater reported subjective cognitive difficulties than other groups, but instead were comparing self to informant report [19, 26], or subjective to objective impairment [25]. The findings that can be drawn from Table 2 are that all four studies evaluating subjective global cognitive impairment reported difficulties [2, 24, 28, 29]. Additionally, all three studies assessing language, memory, and attention difficulties found subjective impairment in all these domains [18, 22, 27]. One study reported similar levels of concerns across all three domains, although much variability depending on the individual item [18]. Armstrong et al. [22] found that more people reported memory problems (~ 50%), followed by language and comprehension difficulties (~ 33%), and finally attention difficulties (27%). Finally, Racine et al. [27], who specifically examined people who underwent awake surgical resection due to tumour location near language, motor, or sensory regions, reported that language difficulties were most prevalent (73–77%), but memory, EF, and attention difficulties were also common. Although less common, participants in this study also reported difficulties with processing speed, visual function, and reading/spelling [27]. The three studies that evaluated EF all reported difficulties [18, 19, 27], with Pranckeviciene et al. [18] finding similar levels of reported EF difficulty to memory, language, and attention.

### Association between self-reported cognition and patient characteristics

None of the included studies reported on the relationship between patient characteristics (i.e. age, socioeconomic status, education, marital status) and subjective cognitive concerns. Hence, this remains an avenue for future investigation.

### Association between self-reported cognition and tumour and treatment characteristics

The associations between self-reported cognition and tumour/treatment characteristics are also currently not well-covered within the literature. None of the studies examined the relationship of time since diagnosis or treatment with self-reported cognition. Further, only two studies provided any information about the relationship between self-reported cognition and other tumour or treatment characteristics. Klein et al. [2] reported that the level of global subjective impairment and subjective communication difficulties were not affected by treatment modality or use of corticosteroids, although both global subjective impairment and subjective communication impairment were related to antiepileptic medications. Schei et al. [28] similarly found corticosteroids did not relate to subjective cognitive impairment, once other variables were accounted, but also found no relationship between antiepileptic medications and self-reported cognition, prior to treatment. Regarding tumour lateralisation, both studies reported worse subjective cognition associated with left-sided tumours [2, 28]. Klein et al. [2] showed that left lateralisation of the tumour only impacted self-reported communication (although note this was the only cognitive domain examined), whereas Schei et al. [28] found that left sided lateralisation more than tripled the likelihood of self-report of any cognitive difficulties. Schei et al. [28] was additionally the only study to investigate tumour size, reporting that greater self-reported cognitive impairment was associated with larger tumour size.

### Alignment between objective assessment and subjective report

As demonstrated in Table 3 10/11 studies also used standardised neuropsychological tests to obtain objective cognitive data. The 10 studies employed 30 different objective tests, with the Trail Making Test [18, 22, 23, 25, 27, 29], Stroop Colour-Word Test [2, 19, 25, 28], Digit Span [19, 22, 25, 27, 29], and Verbal Fluency Test [18, 27, 29] being the most frequently used measures.

**Table 3:** Alignment between self-report and objective assessment and informant report

First author (year)	Alignment between objective assessment and self-report	Alignment between informant and self-report
Armstrong (2012) [22]	NR	Consistent report of symptoms
Braun (2021) [23]	NR	Consistent report of symptoms
Cubis (2019) [24]	Strong alignment	N/A
Donix (2022) [25]	Weak alignment	N/A
Ediebah (2017) [26]	NR	Consistent report of symptoms ^a^
Klein (2001) [2]	Moderate alignment	N/A
Pranckevicience (2017) [18]	Moderate alignment	N/A
Racine (2015) [27]	NR	NR
Schei (2022) [28]	N/A	N/A
van der Linden (2020) [19]	Weak alignment	Inconsistent findings ^b^
Wang (2022) [29]	NR	N/A

^a^Patients with poor cognitive function had lower levels of agreement with informants, compared to patients with normal cognitive function. ^b^Consistent reporting between subjective and informant on the Behavioural Regulation Index of the BRIEF, however, incongruence on the Metacognition Index

Only five [2, 18, 19, 24, 25] of the 10 studies compared objective assessment and subjective reports, with mixed findings, as exhibited in Table 3. Cubis et al. [24] observed a strong correlation between high global self-reported cognitive concerns and lower global objective cognitive status, as assessed by the Brief Test of Adult Cognition by Telephone (BTACT). Similarly, Klein et al. [2] found that global self-reported cognitive functioning was moderately associated with reduced objective attention, processing capacity, and graphomotor speed. Interestingly, Pranckeviciene et al. [18] found moderate associations between subjective and objective memory, and similarly attention, but not other domains, or cross-domain relationships. In contrast, Donix et al. [25] found no relationship between subjective and objective memory. Self-reported EF demonstrated a moderate association with an objective working memory measure, but minimal relationships with objective measures of inhibition and shifting attention [19].

### Alignment between self- and informant-report

Five of the studies also included informant report, with four of these providing information regarding the consistency between self and informant reports, as shown in Table 3. Concerns about cognition were consistent between informants and patients in relation to memory and communication in two studies [22, 26]. In contrast, there were contradictory findings on the consistency between subjective and informant reports of EF. The report of EF deficits using the BRIEF-A and the informant report version, the BRIEF-IR, indicated a high degree of agreement in one study [23]. Comparatively, a separate study demonstrated good alignment between subjective and informant reports on the BRIEF Behavioural Regulation Index, but differences on the Metacognition Index, with patients reporting more difficulties in this area than their informants [19]. The metacognition index refers to the more cognitively laden EF skills of working memory, self-monitoring, planning, and organisation. Conversely, the Behavioural Regulation Index assesses the more readily observed, behaviourally manifested EF, such as inhibitory control, emotional regulation, and behavioural initiation.

### Quality analysis and risk of bias

The results of critical appraisal revealed that the majority of studies employed methodologies that were both valid and reliable. One study presented a notable limitation [2], as cognitive data were collected across different locations for participants (i.e. 73% had cognitive assessments at home), potentially decreasing reliability in measurement. Of the 11 studies reviewed, five were deemed to have insufficient sample sizes [18, 19, 25, 27, 29], recruiting fewer than 65 participants, including three studies with sample sizes under 25 [25, 27, 29], which limits the generalisability of the results. Furthermore, three studies lacked an appropriate sample frame: one study imposed exclusion criteria (e.g. differing histology, infratentorial tumour location, survival times under 12 months, or recurrent glioma) that impacted the sample’s alignment with the target population [25]; another exclusively recruited participants from a cognitive rehabilitation trial [19]; and a third reported that 63% of participants had frontal tumours [29], further impacting the representativeness of the sample to the target population. Reporting on response rates was inconsistent, with four studies failing to provide any information [23–25, 27], and one study offering comprehensive response rate data [18], but omitting details on participant attrition. Lastly, one study demonstrated inappropriate statistical coverage [27], with its data after attrition being skewed towards younger participants (i.e. 83% under 50 years old), thus failing to adequately reflect the broader primary brain tumour (PBT) population. The specific results for each study are displayed in Table 4.

**Table 4:** Results of quality assessment

First author (year)	Appropriate sample frame	Participants sampled appropriately	Adequate sample size	Sufficient detail of participants and setting	Statistical coverage of the population	Valid methods	Reliable measurement	Appropriate statistical analysis	Response rate detailed
Armstrong (2012) [22]	** + **	** + **	** + **	** + **	** + **	** + **	** + **	** + **	** + **
Braun (2021) [23]	** + **	** + **	** + **	** + **	** + **	** + **	** + **	** + **	**-**
Cubis (2019) [24]	** + **	** + **	** + **	** + **	** + **	** + **	** + **	** + **	**-**
Donix (2022) [25]	**-**	**-**	**-**	** + **	** + **	** + **	** + **	** + **	**-**
Ediebah (2017) [26]	** + **	** + **	** + **	** + **	** + **	** + **	** + **	** + **	** + **
Klein (2001) [2]	** + **	** + **	** + **	** + **	** + **	** + **	**-**	** + **	** + **
Pranckeviciene (2017) [18]	** + **	**-**	**-**	** + **	** + **	** + **	** + **	** + **	**-**
Racine (2015) [27]	** + **	** + **	**-**	** + **	**-**	** + **	** + **	** + **	**-**
Schei (2022) [28]	** + **	** + **	** + **	** + **	** + **	** + **	** + **	** + **	** + **
van der Linden (2020) [19]	**-**	** + **	**-**	** + **	** + **	** + **	** + **	** + **	** + **
Wang (2022) [29]	**-**	** + **	**-**	** + **	** + **	** + **	** + **	** + **	** + **

## Discussion

This systematic review sought to investigate subjective cognitive concerns reported following PBT. Specifically, we aimed to document the most commonly reported concerns, whether they vary with patient, tumour, or treatment characteristics, and to determine the congruence with objective cognition and informant report. Findings from the 11 studies included revealed self-reported impairment in overall cognition, as well as in most domains assessed, with some evidence that difficulties with language, memory, attention, and EF were most common. Evidence from two studies suggested left-sided tumours were associated with greater subjective cognitive impairment, and one study reported an association with tumour size. Alignment between objective and subjective cognition was variable, whereas alignment between self and informant reports was generally good.

We had difficulty determining the most commonly impaired subjective domains, as few studies compared the relative frequency or importance of cognitive domains. There was a lack of comprehensive assessment across multiple cognitive domains, with many studies only focusing on a targeted domain, or only considering a global cognitive measure. Additionally, there was considerable variation in the degree of subjective cognitive assessment, ranging from a single question to more comprehensive questionnaires per domain. Future studies conducting a comprehensive and detailed comparison of subjective cognition across domains is warranted to better characterise these patient-reported outcomes.

While determining the associations or predictors of worse subjective cognition is an integral question for proactive patient care, this was rarely examined in the literature. No studies examine the relationship between subjective cognition and patient or treatment characteristics. Only two studies examined the relationship with some tumour characteristics [2, 28], with both reporting worse subjective cognition related to left lateralisation, and no relationship to corticosteroid use. One of the two studies examining tumour characteristics also found evidence that larger tumours related to worse cognition [28]. Further, these studies reported differing results regarding the impact of anti-epileptic medications. While a negative impact is consistent with the adverse effects of antiepileptic medications on cognition reported in other populations [32, 33], there is currently insufficient literature on the impact following PBT. Indeed, this would also need to be considered in relation to the impact of uncontrolled seizures on cognition. It is also likely that patient, tumour, and treatment characteristics have different impacts at different times post-diagnosis, as well as interact with one another to impact subjective cognition. A comprehensive, large, longitudinal study would be needed to properly evaluate these interactions. Although, it is possible that the effects of tumour lateralisation and other tumour characteristics might be more commonly reported in studies examining changes in language after a brain tumour. Studies focusing on specialised cognitive functions, such as language, often align with investigations into tumour characteristics like lateralisation or the involvement of specific brain regions [34]. By not including these terms, we likely excluded research that could provide valuable insights into the relationship between tumour characteristics and their impact on specialised cognitive domains. This omission may have also limited the review’s ability to capture the full scope of cognitive outcomes and led to an incomplete understanding of the cognitive challenges faced by this population. Therefore, it is recommended that a more targeted approach incorporating both broad and specific terms could ensure a more comprehensive account within future literature reviews.

Regarding the alignment between subjective cognitive concerns and objective cognition, findings were mixed, with one study reporting strong alignment, two moderate alignment, and two weak alignments. The strongest alignment occurred when comparing a global subjective and objective cognitive measure [24]. In terms of individual domains, findings were mixed with moderate alignment in some studies on memory, attention, EF, and speed, but other studies reported no alignment on memory and EF. These findings perhaps reflect the broader literature demonstrating mixed alignment between subjective and objective cognition in ageing [35], epilepsy [36], schizophrenia [37], multiple sclerosis [38], and cancer [39].

Patients with reduced insight following a PBT often have more severe cognitive impairment and hence perform poorly on objective measures. Conversely, patients with less cognitive impairment can be more acutely aware of their cognitive changes and may report greater subjective impairment [40, 41]. Indeed, those with greater insight into their cognitive change could have greater distress, impacting their ratings of cognitive impairment. One of the limitations of our review was that we did not systematically consider the association of subjective cognition in PBT with any psychological variables, such as mood or distress. Despite this, two of the included studies supported an association [2, 25]. Furthermore, other studies in PBT [42], as well as other conditions [34, 36–39], suggest distress or low mood is often a contributor to low ratings of subjective cognition. Future studies should ensure the inclusion of psychological variables when examining subjective cognition.

In contrast to the varied alignment between subjective and objective cognition, our review found considerable agreement between self and informant reports. There was some variability in ratings of EF, particularly for meta-cognitive rather than behavioural aspects of EF. Note, two studies reported less congruence in self and informant reports for patients with lower overall function [22, 26]. This implies that patients generally do have insight into their cognitive difficulties. It further suggests that informant reports represent a good approximation of subjective cognitive concerns for patients with primary brain tumours. These findings support the use of informant reports in addition to subjective reports, as they can contribute to a better understanding of the impairment.

The quality analysis highlighted that, while the studies were methodologically sound for the most part, some quality issues were revealed with implications for future research. A recurring issue was small sample sizes, with nearly half of the studies (5/11) recruiting fewer than 65 participants. This is likely reflective of several factors, including the rarity of PBT which constitutes less than two percent of all cancer diagnoses [43], the overall poor prognosis of PBT, and the resource-intensive nature of either longitudinal or neuroimaging research, which inherently limits participant numbers. While these constraints are understandable, they also emphasise the need for wider-reaching recruitment strategies, multi-site collaborations, or pooled data approaches to enhance sample representativeness. Along with the studies that had more restricted samples [19, 25, 29], or were skewed towards younger people [27], this issue leads to reduced representativeness of the studies regarding the general population of people with PBT. Another quality issue was the inconsistent reporting of response and attrition rates, with four studies failing to document response rates and one study providing only detailed response rate data without attrition information. Given the significant health and functional impacts of brain tumours, understanding patterns of attrition is beneficial for designing longitudinal studies in order to account for potential dropouts. Future research should additionally systematically report these factors to identify whether certain demographic or clinical characteristics make participation more or less likely, ultimately improving study retention and representation. The overall variability in study quality underscores a broader challenge in this research area—the need for more standardised methodologies and reporting practices to strengthen the evidence base and guide clinical decision-making. Addressing these limitations will be essential for improving the robustness and applicability of future research in neuropsychological rehabilitation for individuals with brain tumours.

Our review of the literature demonstrates that self-reported cognitive concerns have corroborative validity. Therefore, clinicians can strengthen their understanding of patients’ cognitive challenges and gain important insight into which cognitive domains are most impacted through assessing self-reported cognitive concerns. Indeed, this may be used in situations where a comprehensive neuropsychological assessment is not available. One of the limitations of our review was that we did not identify any instruments that were comprehensive in assessing the main cognitive domains sufficiently while remaining short enough to be feasible to administer during clinic visits with PBT patients who often have substantial fatigue. The FACT-COG [31] has strong psychometrics [44] and is feasible to administer but only provides an overall score, rather than identifying the specific cognitive domains impacted. Future research may look at whether the FACT-COG can be validly used to assess and compare subjective cognition across various domains. Alternatively, new measures may need to be developed and validated.

Collectively, these findings highlight the incidence, diversity, and complexity of subjective and informant-reported cognitive impairment related to PBT. In order to improve patient quality of life, it is important that subjective cognitive deficits are assessed systematically. This will enable the development and provision of appropriate interventions to improve patient experience and functioning in everyday life.

## Data Availability

No datasets were generated or analysed during the current study.
